# No effect of the short-term learning of object trajectories on multisensory perception within the peripersonal space

**DOI:** 10.3758/s13414-026-03230-x

**Published:** 2026-02-11

**Authors:** Daisuke Mine, Takuji Narumi

**Affiliations:** Graduate School of Information Science and Technology, The University of Tokyo, Bunkyo-ku Hongo 7-3-1, Tokyo, Japan

**Keywords:** Crossmodal congruency effect, Multisensory interaction, Peripersonal space, Spatial representation, Body representation

## Abstract

Peripersonal space, the space immediately surrounding the body, functions as an interface between the self and the external environment. In interactions with dynamic environments, not only bodily information but also accurate estimation of the dynamics of external objects is required. While numerous studies have demonstrated that real-time estimation of object dynamics, based on bottom-up mechanisms, modulates peripersonal space representation, little evidence supports the involvement of top-down knowledge about object dynamics in shaping peripersonal space. In the present study, we conducted two experiments to examine whether pre-learned statistical regularities of object trajectories influence performance in a visuo-tactile task designed to assess multisensory perception within the peripersonal space. Participants first learned the statistical tendencies of visually presented object movements, and subsequently performed a visuo-tactile reaction task. Across both experiments, we observed no significant changes in task performance as a function of the learned object dynamics. These findings call for a reconsideration of the role of top-down knowledge in the modulation of peripersonal space representation.

## Introduction

Tactile perception is affected by visual or auditory stimuli presented within the space immediately surrounding the body, called the “peripersonal space.” Behavioral studies have shown that faster tactile reaction time is observed when task-irrelevant visual/auditory stimuli are presented within the peripersonal space than when they are presented outside of the peripersonal space (for review, see Serino, 2019). Straka, Noel, and Hoffmann (2022) proposed a Bayesian computational model that can explain a wide variety of characteristics of peripersonal space reported in previous studies by computing the impact probabilities between our body and external objects in the near future. Many previous studies have suggested the idea that peripersonal space has a function that supports the interaction between our body and dynamic objects (Hunley & Lourenco, 2018; Serino, 2019). To achieve desirable interactions with a dynamically changing external environment, both effective control of our own body and accurate prediction of object movement are essential, as neither can be neglected. A large amount of research in humans has revealed that various types of modulation of our action capability change peripersonal space representation (e.g., tool use: Longo & Lourenco, 2006; Serino, Bassolino, Farnè, & Làdavas, 2007; performing specific actions: Noel et al., 2015; spatial modulation of body parts: Mine & Yokosawa, 2021a, 2021b; modulation of visual feedback of our movement: Mine & Yokosawa, 2022; Mine & Narumi, 2024). On the other hand, how much the effect of prediction of objects’ movements depends on peripersonal space is still not completely clear.

Movement information about objects can be categorized into real-time estimation and prior knowledge. Real-time estimation involves a bottom-up system estimating the position, speed, and direction of movement of an external object while it is in motion and predicting the future position of the object. It has been shown that the velocity of an approaching stimulus affects peripersonal space representation, typically higher velocity expands peripersonal space, in neural (Fogassi et al., 1996) and behavioral (Noel, Blanke, Magosso, & Serino, 2018) studies. Another behavioral study using dynamic auditory stimuli found that the audio-tactile interaction effect changes according to the movement direction of auditory stimuli (Canzoneri, Magosso, & Serino, 2012). These authors showed that the tactile reaction time speeded up and was more sensitive to the location of auditory stimuli when auditory stimuli are heading toward the body than when receding from the body. These are the examples of evidence of the effect of real-time estimation on the multisensory perception within peripersonal space. In addition, a neural study in monkeys showed that tactile sensitivity was enhanced by predicted location and time of impact between the body and looming objects (Cléry et al., 2015). A neural study in humans also provided evidence suggesting that reactions to looming stimuli depend highly on bottom-up reflexive mechanisms (Makin, Holmes, Brozzoli, Rossetti, & Farnè, 2009a, 2009b). The study revealed that the suppression of motor-evoked potentials in participants’ hands occurred within a very short time, approximately 70 ms after the appearance of the looming stimuli. It is widely accepted that an involuntary bottom-up system capturing objects’ dynamics is an essential component underlying human reactions to looming stimuli.

Prior knowledge is involved in our comprehension of the potential trajectories an object would follow, derived from learned statistics of the objects’ dynamics or from estimation based on lifelong experiences in our daily lives. For example, we know that trains run on tracks, and it is highly unlikely that trains would go where tracks are not laid. While real-time estimation is supported by a number of studies, there are a few studies investigating the top-down effect of prior knowledge of the trajectories objects possibly follow on the peripersonal space representation. The study by Huijsmans, de Haan, Müller, Dijkerman, and van Schie (2022) is one of the very few demonstrating the effect of the contextual knowledge of the impact between the body and external objects manipulating trajectories the objects would follow. In their study, computer-simulated insects moved along two different paths, one of which was the collision path that ends at the participant’s hand, and the other was the near-miss path which misses the participant’s hand by several centimeters. Participants in their study showed faster response to the tactile stimuli when the insects moved along the collision path than along the near-miss path, especially when insects were at locations near their hands. This result suggested that contextual prediction of the object trajectory affects multisensory perception within the peripersonal space. In that experiment, the paths the objects followed had a frame of solid lines and were visible throughout the task. The visualization of the paths provides explicit cues about possible trajectories the objects could take. Moreover, there is a possibility that the visualized path could attract participants’ attention to either path, especially to the path the participant’s hand is placed on. However, the paths looming objects follow are not always visually presented. For example, when playing baseball, a batter predicts the type of pitch and its trajectory and adjusts their motor intentions accordingly based on the predicted trajectory. In this way, humans can prepare their movements and interact with objects even when visual cues about an object’s motion are not explicitly presented. It is essential to investigate how the multisensory perceptual system within the peripersonal space functions in such interactions.

In the present study, participants learned the trajectories of two differently colored spheres in which the color related to its trajectory: one sphere approached the participants, while the other moved away in a different direction. Then, participants performed a visual-tactile reaction task using two colored spheres as visual stimuli. In two experiments, we assessed if pre-learned indirect information of the objects’ trajectories affects the perception within the peripersonal space. Previous studies have shown that the short-term training of the statistical tendency of objects’ moving speed changes the prediction of the location of moving stimuli (Kwon & Knill, 2013; Makin, Stewart, & Poliakoff, 2009a, 2009b). They revealed that humans can extrapolate the location of moving objects from pre-learned statistics of an object’s speed without direct visual cues about the object’s dynamics. Given peripersonal space representation is strongly related to the interaction with external objects, it is possible that participants would show greater facilitation on tactile reaction time in the presence of visual stimuli when the visual stimuli would collide with their body. On the other hand, if the main function of multisensory perception within peripersonal space in the interaction is detecting the presence of objects that might be a candidate for the interaction, fast processing of the objects’ location and their rough movement based on bottom-up information is more important than relatively slow estimation of accurate dynamics of the objects. If this is the case, top-down knowledge about objects’ trajectories do not have a strong effect on multisensory perception within peripersonal space.

## General methods

### Participants

We recruited 56 healthy participants as paid volunteers: 28 participants (15 women; mean age = 26.3 years, SD = 7.5 years) for Experiment 1 and 28 participants (11 women; mean age = 26.7 years, SD = 8.6 years) for Experiment 2. The sampling plan was preregistered (10.17605/OSF.IO/TQFCR). We calculated the sample size of each experiment based on a desired power of 0.95 and the assumed effect size (*dz* = 0.75). The required sample size was 26; however, in order to counterbalance the order of the tasks and the conditions, we recruited 28 participants for each experiment. All participants were right-handed and had normal or corrected-to-normal stereo vision.

All participants provided written informed consent before participating in the experiments. The local ethics committee approved the experiments and procedures described below. The experiments were conducted in accordance with the principles and guidelines of the Declaration of Helsinki.

### Apparatus and stimuli

Participants viewed a virtual environment through a head-mounted display (HMD) (META Quest 2, displaying a stereoscopic image with a resolution of 1,832 × 1920 and a field of view of 90°). A virtual world was developed using Unity3D and run on a Windows PC (Alienware M15 R4, Intel Core i7-10870H, 16 GB RAM, and NVIDIA GeForce RTX 3080). A vibrator (HAPTIC Reactor AFT14, ALPS ALPINE Co., Ltd.) was used to deliver tactile inputs to participants’ right middle fingers.

## Experiment 1

### Procedure

Participants sat at one end of a table in the lab space. Before the experiment started, the vibrator was attached to the participant’s right middle finger. Participants were then instructed to adjust the fit of the HMD. Participants placed their right hand on the table, 30 cm in front of their body, and kept it there throughout the experiment. Participants performed three tasks: the unisensory tactile reaction task, the learning task, and the visual-tactile reaction task. Half of the participants performed the unisensory task before the other tasks, and the other half performed the unisensory task after the other tasks. The visual-tactile reaction task was always preceded by the learning task.

The unisensory task was performed to assess the baseline of tactile reaction time for each participant. In this task, participants were required to react to the tactile stimulation on their right middle finger by pressing a button. When a trial started, a white sphere was presented for 1 s at 220 cm in front of the participant. Tactile stimuli were administered 0.8 s or 1.8 s after the sphere disappeared. Participants pressed a button when they felt a tactile stimulation at their right middle finger. After a 2-s interval, the next trial started. In this task, the participants performed 120 trials: two timings of tactile stimulation × 24 repetitions and 24 catch trials (no tactile stimuli).

**Fig. 1:**
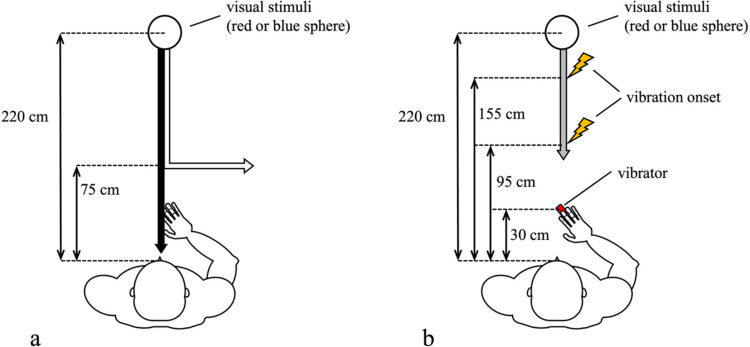
Schematic view of Experiment 1. In the learning task, participants repeatedly observed approaching visual stimuli (red or blue sphere). The sphere’s color is related to its trajectory: one approached participants’ bodies, and another changed direction 75 cm from participants and moved away from them (**a**). In the visual-tactile reaction task, participants reacted to the tactile stimulation on their right middle finger while ignoring visual stimuli (the red or blue sphere). The tactile stimulation was presented when visual stimuli passed either of two locations, 95 cm (inside the peripersonal space) or 155 cm (outside the peripersonal space) from participants (**b**)

In the learning task, participants repeatedly observed moving visual stimuli, a red- or blue-colored sphere, and were instructed to remember the movement trajectory of the visual stimuli. Each colored sphere moved following different paths: one directly approached participants’ bodies from the front direction of the participants (collision path), and the other followed the same trajectory in the middle of the movement and turned right or left at the location 75 cm in front of participants (no-collision path) (Fig. 1a). The combinations of the colors and paths were counterbalanced across participants. When a trial started, a sphere of either color appeared at 220 cm in front of participants. One second after the sphere appearance, the sphere started moving at a velocity of 75 cm/s and continued moving for 3 s following each of the paths. Three seconds from the movement onset, the sphere disappeared, and the next trial started. In 10% of the trials, the sphere flashed 2 s after the sphere started moving. Participants were required to push a button with their left middle finger when they noticed the flashing to make sure that participants thoroughly observed the sphere. The learning task consisted of ten blocks of 20 trials each. On average, participants took almost 40 min to complete the learning task.

In the visual-tactile reaction task, participants were required to press a button with their left middle finger as quickly as possible when they felt tactile stimuli administered on their right middle finger while ignoring approaching visual stimuli. This task aimed to examine whether the top-down knowledge of the association between the visual stimuli’s colors and their future trajectories, acquired in the learning task, would influence tactile reaction time. Visual stimuli, the same red or blue spheres used in the learning task, appeared at 220 cm in front of participants, and 1 s after, the sphere started moving at a velocity of 75 cm/s as in the learning task. Tactile stimuli were administered 0.8 s or 1.8 s after visual stimuli started moving. The onset of the tactile stimulation corresponded to the spatial dimensions of the visual stimuli at 155 cm (far trials) or 95 cm (near trials) from participants, respectively (Fig. 1b). It has been suggested in a previous study (Mine & Yokosawa, 2022) that when visual stimuli are present at a position around 95 cm from the body, this reflects the nature of perception within the peripersonal space, and when the visual stimuli are at a position around 155 cm from the body, this reflects the nature of perception out of the peripersonal space. Visual stimuli disappeared 200 ms after tactile stimulation, which occurred before the location where the two spheres diverged in the learning task. Therefore, in this task, the two colored spheres always followed exactly the same visible trajectory, even though participants had learned that they would later diverge into collision and no-collision paths. After a 2-s interval, the next trial started. Trials in which the sphere’s color matched the color associated with the collision path in the learning task were defined as Collision trials. Conversely, trials in which the sphere's color matched the color associated with the no-collision path in the learning task were defined as No-Collision trials. The participants performed 120 trials: two visual stimuli colors [red or blue] × visual stimuli positions [near or far] × 24 repetitions and 24 catch trials (no tactile stimuli).

### Statistical analysis

As preregistered (10.17605/OSF.IO/TQFCR), a paired *t*-test was conducted on the mean visual facilitation effect when visual stimuli were near the body. The visual facilitation effect was defined by the difference between the tactile reaction time in the unisensory task and in the visual-tactile reaction task. Outliers were defined as reaction times that exceeded 1,000 ms or three times the standard deviation from each participant’s mean reaction time for each condition; these were excluded from the following analyses. All the relevant analyses were conducted using the statistical software package R.

### Results and discussion

**Fig. 2:**
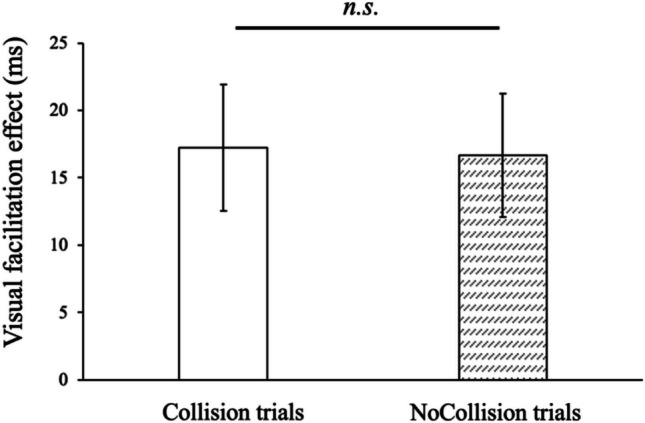
Visual facilitation effect in Experiment 1. Positive values mean smaller reaction times in the visual-tactile task than in the unisensory task. Error bars indicate standard errors

The results of Experiment 1 are illustrated in Fig. 2. A paired *t*-test shows no significant effect of the paths on the visual facilitation effect when visual stimuli were located near the body (*t*(27) = 0.39, *p* =.70, *dz* = 0.074). This result suggests that short-term learning of the pair of object trajectories and their color was not enough to change the multisensory perception within the peripersonal space.

The following analyses are exploratory ones which were not preregistered. We conducted a two-way analysis of variance (ANOVA) on the visual facilitation effect, with visual‑stimulus position (95 vs. 155 cm) and path (collision vs. no-collision) as factors, to test whether the visual facilitation effect properly occurred when visual stimuli were presented near the body. The ANOVA revealed a significant main effect of the visual stimuli position (*F*(1, 27) = 51.14, *p* <.001, ${\eta }_{p}^{2}$=.65), suggesting visual stimuli at 95 cm induced a larger visual facilitation effect than visual stimuli at 155 cm, which is consistent with a previous study (Mine & Yokosawa, 2022). No main effect of path and no interaction were observed. Moreover, we calculated a Bayes factor to assess the strength of evidence supporting a null hypothesis in the comparison between the collision and the no-collision path conditions when visual stimuli were located near the body. A Bayesian analysis (using a wide prior, Cauchy distribution with location = 0 and scale = 1) revealed moderate evidence for the absence of a difference between the collision path condition and the no-collision path condition (BF_01_ = 6.37).

These results did not indicate a significant effect of prior knowledge about the trajectories of spheres. However, there is a possibility that participants did not concentrate on observing spheres’ trajectories in the learning task of this experiment. In this task, since participants only needed to press a button when a sphere flashed regardless of whether the sphere would collide with their body, they could observe spheres in a distracted manner without dedicating much attention to their trajectory or their color. To address this issue, in Experiment 2 we modified the learning task for participants to focus more on the judgment of collision and remembering the color of the pair of spheres and their trajectory.

## Experiment 2

### Procedure

**Fig. 3:**
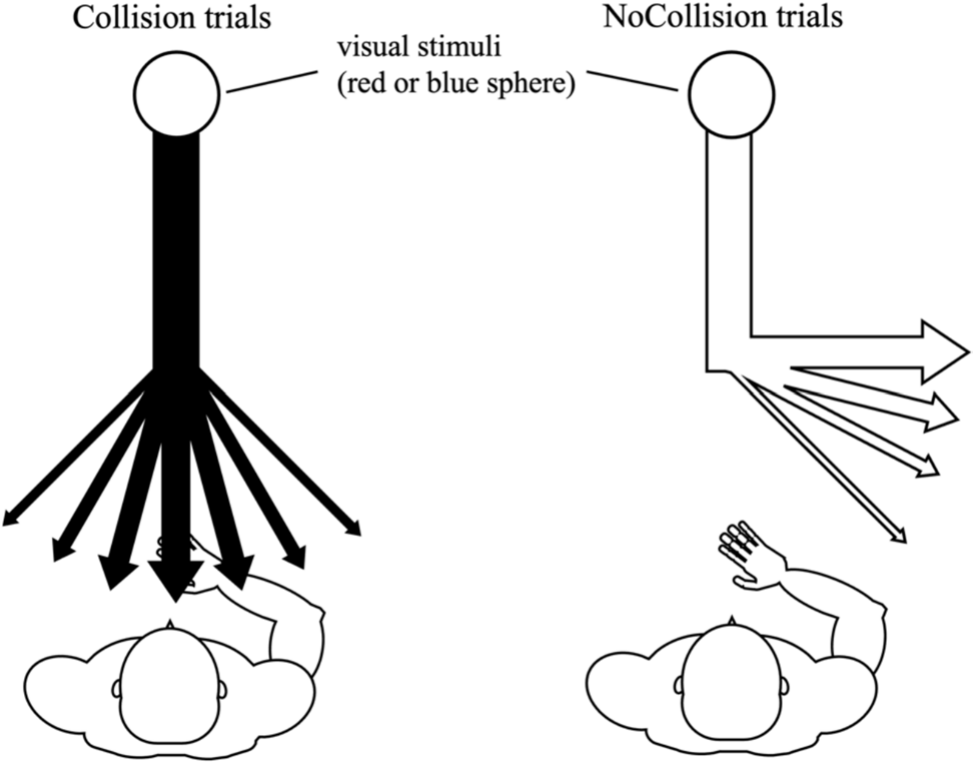
Schematic view of the learning task in Experiment 2. Spheres probabilistically changed their direction 75 cm from the participants. In the collision trials, the sphere approximately approached the participant’s body. In the No-Collision trials, the sphere moved away from the participant’s body. Participants were instructed to judge whether the sphere would collide with their body by pressing keys with their left index or ring finger

Twenty-eight paid volunteers participated in this experiment and performed the three tasks, which followed almost the same procedures as those outlined in Experiment 1, with the following exception. In the learning task, participants were required to observe a red or blue sphere and judge whether or not the sphere would collide with their body by pressing either of two buttons. This task consisted of six blocks. In half of the blocks, participants were instructed to press one of two buttons using either their left index or ring finger, depending on the color of the sphere, when they judged that the sphere would collide with their body. In the remaining blocks, they were instructed to press the corresponding button depending on the color of the sphere when they judged that the sphere would not collide with their body. The mapping between each color and the corresponding button was consistent within each participant, while the color-button position pairing was counterbalanced across participants. Participants conducted each block type alternatively. To increase task complexity and ensure participants accounted for the sphere’s trajectory, each sphere probabilistically changed direction with a certain tendency at a point 75 cm in front of them. One color of the sphere moved approximately toward the participant’s body (collision path: 40% at 0 degrees, 30% at 15 degrees, 20% at 30 degrees, and 10% at 45 degrees, with 0 degrees indicating a sphere moving directly toward the participant and positive values indicating the rightward direction from the participant’s perspective), while the other color of the sphere moved away from the participant's body (no-collision path: 40% at 90 degrees, 30% at 75 degrees, 20% at 60 degrees, and 10% at 45 degrees) (Fig. 3). Through this task, participants learned the association between the color of the sphere and the tendency of its trajectory. Procedures of the other tasks were exactly the same as in Experiment 1.

### Results and discussion

**Fig. 4:**
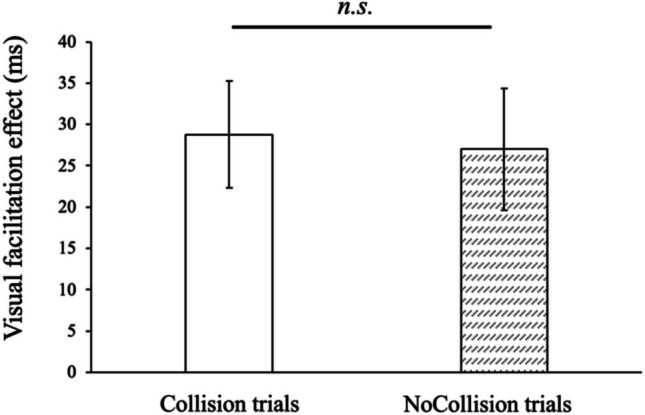
Visual facilitation effect in Experiment 2. Positive values mean smaller reaction times in the visual-tactile task than in the unisensory task. Error bars indicate standard errors

The results of Experiment 2 are illustrated in Fig. 4. We conducted the same analyses as in Experiment 1. A paired *t*-test revealed no significant effect of the paths on visual facilitation effect when visual stimuli were located near the body (*t*(27) = 0.83, *p* =.42, *dz* = 0.16). As exploratory analyses, a two-way ANOVA showed a significant main effect of the location of visual stimuli (*F*(1, 27) = 95.6, *p* <.001, ${\eta }_{p}^{2}$ =.78), suggesting the presence of a visual facilitation effect near the body as in Experiment 1. No other significant effects were observed in this analysis. Moreover, we calculated a Bayes factor to assess the strength of evidence supporting a null hypothesis in the comparison between the collision and the no-collision path conditions when visual stimuli were located near the body. A Bayesian analysis (using a wide prior, Cauchy distribution with location = 0 and scale = 1) revealed moderate evidence for an absence of difference between the collision path condition and the no-collision path condition (BF_01_ = 4.93). The results showed the same tendency as in Experiment 1. Prior knowledge about object trajectories did not influence the visual facilitation effect, even after the learning task which required understanding the association between the sphere’s color and its trajectory.

## General discussion

This study aimed to investigate the effect of prior knowledge about the trajectory of looming objects on multisensory perception within peripersonal space. The results of the two experiments did not indicate the presence of the effect of prior knowledge about objects’ trajectories on visual facilitation effect. These results partially contradict previous findings showing that the difference in destinations of the paths’ visual stimuli would follow affected visual facilitation effect (Huijsmans et al., 2022). Huijsmans et al.’s study and ours both investigated the effect of information about the trajectory visual stimuli would follow, which is not directly obtained from observing the movement of the visual stimuli. The main difference between Huijsmans et al.’s study and ours is the visualization of the paths. Two main possibilities can be proposed as likely reasons for the differences in results between their study and the present study. One is that the visual facilitation effect relies strongly on contextual information regarding an object’s path that is presented visually at a given moment. In the visual-tactile reaction task of the present study, participants had to predict the trajectory from the visual stimuli’s color based on the pre-learned knowledge of the combinations between the colors and trajectories. There was no direct visual clue of the object’s trajectory in the environment. On the other hand, in Huijsmans et al.’s study, the visualized paths can offer a direct clue for predicting the future location of visual stimuli. Prior knowledge that an object never collides with one’s body is insufficient to alter the visual facilitation effect. It is hypothesized that a change in the visual facilitation effect occurs only when such knowledge is integrated with the visual information presented at the moment.

Another account is the object-based attentional effect. It is well known that human attention does not always spread in a space-based manner, in which attention spreads evenly from its center to the outer space referring to a spotlight, but it can be captured by a specific object. A previous study showed that detection of a visual target placed on the same object on which a prior cue had been placed was faster than a visual target placed out of the object even when the distance between the prior and the targets was exactly same (Egly, Driver, & Rafal, 1994). Analogous to visual attention, tactile attention can also be object based (Gillmeister, Adler, & Forster, 2010). Moreover, it has been reported that object-based attention occurs across modalities (cf. Busse, Roberts, Crist, Weissman, & Woldorff, 2005, and Turatto, Mazza, & Umiltà, 2005, suggesting evidence not for visual-tactile but for audio-tactile object-based attentional effects). It is possible that the two visualized paths might function as distinct objects, and each path, one ending at the participant’s hand and the other missing the participant’s hand, captured object-based attention when the visual stimuli entered one of the paths. If this is the case, the difference in tactile reaction time between the collision condition and the near-miss condition, observed in Huijsmans et al.’s study, might be accounted for by a benefit of attention captured by a collision path and/or a cost of switching attention from the attended location captured by a near-miss path. In this explanation, since the presence of the effect of top-down knowledge on the trajectory of visual stimuli is not required, the results of both our study and the previous one can be adequately accounted for. Since there is no direct evidence of the visual-tactile object-based attention, this explanation does not allow for an immediate conclusion that human reactions to approaching stimuli are solely based on bottom-up mechanisms and are out of control of top-down knowledge. We suggest that the potential influence of top-down processes on these perceptual systems requires further detailed investigation in future studies.

Another possible explanation for our results is that the visual stimuli in our study did not provide participants with a motivation to prevent themselves from colliding with the visual stimuli. Some previous studies have suggested that peripersonal space and related visual perceptual systems are more strongly affected by fearful or noxious visual stimuli than neutral ones (Huijsmans et al., 2022; Taffou & Viaud-Delmon, 2014; Vagnoni, Lourenco, & Longo, 2012; Vagnoni, Lourenco, & Longo, 2015; Zadra & Clore, 2011). A recent computational model explaining the various features of peripersonal space incorporates the evaluation of the false negative representing the penalty of not predicting the impact when it occurs (lower evaluation of the false-negative results in a smaller area of peripersonal space) (Straka et al., 2022). Given that the anticipated intensity of negative consequences from an impact between a visual stimulus and the body is a key factor influencing the visual facilitation effect, it may be reasonable that judgment of the paths of visual stimuli (colliding or not) had little influence in the present experiment, which employed neutral visual stimuli unlikely to produce substantial negative effects upon impact. Fearful emotions toward looming visual objects and the anticipation of negative consequences upon impact play a crucial role in survival, particularly in protecting one’s own body. However, reactions to looming stimuli do not always involve defensive intention. Moreover, Huijsmans et al. (2022) did not report the interaction effect between the fearfulness of visual stimuli and the paths visual stimuli would follow. In their study, the participants showed a faster tactile reaction time when they knew the visual stimuli would collide with their hand than when they knew the visual stimuli would miss their hand regardless of whether visual stimuli were fearful (a spider) or neutral (a butterfly). Therefore, the lack of fearfulness of the visual stimuli does not explain the difference in the results of our study and the previous one.

Of course, there is a possibility that our learning task was not enough for participants to construct the association between the colors of objects and their trajectories (colliding or not). Certainly, the learning task was relatively short (almost 40 min). However, we think this view is not necessarily likely because the difference between the two types of trajectories was quite obvious even when there was probabilistic variance. Moreover, in Experiment 2, in which participants placed more attention on the relationship between the spheres’ colors and their tendency for collision with their body during the learning task, the effect of the paths remained non-significant, and its effect size was as small as in Experiment 1. Another limitation was that we adopted only two spatial locations of visual stimuli to shorten the time of the experiments. The visual-tactile reaction task could not be much longer because the association between the color and the trajectory which the participants acquired in the learning task might disappear if the reaction task took a long time.

One might argue that our choice of visual stimulus distances did not capture the multisensory perceptual system within peripersonal space. We adopted 95 cm from the body (65 cm from the right hand) as “inside” peripersonal space, following Mine and Yokosawa (2022), who employed a similar experimental paradigm. It is well established that the extent of peripersonal space varies across studies: some studies reported shorter distances, for example around 47 cm (Kandula, Van der Stoep, Hofman, & Dijkerman, 2017) or between 42 cm and 63 cm (Serino et al., 2015). Importantly, studies reporting shorter distances were conducted in real environments, whereas those reporting longer distances were conducted in virtual environments (Mine & Yokosawa, 2022) or mixed-reality environments (Serino et al., 2018). Egocentric distance is known to be underestimated in virtual environments compared with real ones, which is a phenomenon called distance compression (for review, see Renner, Velichkovsky, & Helmert, 2013). If visual stimuli are perceived as closer than they actually are, it is reasonable that greater peripersonal space boundaries are observed in virtual environments. Taken together with results from comparable studies in virtual and mixed-reality contexts, our choice of visual stimulus distances was suitable for assessing perceptual features within the peripersonal space. The present study aimed to examine how prior knowledge about whether an approaching object would collide with the body influences the visual facilitation effect within peripersonal space. Future research may need to employ paradigms with a greater number of visual-stimulus positions in order to clarify more precisely how prior knowledge shapes the gradual representation of peripersonal space. However, increasing the number of stimulus positions inevitably increases the number of trials, which raises practical concerns. Longer experiments in virtual-reality settings may impose additional burdens on participants, potentially affecting performance, and the prior knowledge acquired before the visual-tactile reaction task may diminish over time. These factors must be carefully considered in designing follow-up studies.

## Conclusion

The present study aimed to assess the effect of top-down knowledge of object trajectories on multisensory perception within the peripersonal space. The results of two experiments did not support the presence of a top-down effect. These findings align with numerous studies indicating a strong reliance of multisensory perception within the peripersonal space on bottom-up mechanisms but contradict one recent study. While some research suggests that humans utilize pre-learned top-down information to extrapolate object movement and prepare sensorimotor responses for interaction (Kwon & Knill, 2013; Makin, Stewart, & Poliakoff, 2009a, 2009b), evidence for a top-down effect on peripersonal space representation remains limited. Importantly, however, the present findings do not rule out the possibility that top-down knowledge can influence perception within the peripersonal space under different conditions. In particular, the present study focused only on top-down knowledge directly related to objects’ dynamics, whereas more semantic forms of top-down information are known to affect the prediction of time to collision which is strongly related to peripersonal space representation (Vagnoni, Lingard, Munro, & Longo, 2020). Future research should investigate the extent and boundary conditions under which various types of top-down knowledge contribute to multisensory perception within the peripersonal space, using stricter control of task variables to precisely characterize when and how such effects emerge.

## Data Availability

The datasets generated and/or analyzed during the current study are available from the corresponding author upon reasonable request.

## Code availability

The codes used during the current study are available from the corresponding author upon reasonable request.

## Declarations

### Open practices statement

The data that support the findings of this study are not openly available and are available from the corresponding author upon reasonable request. None of the materials for the experiments reported here are available. Experiment 1 was preregistered (10.17605/OSF.IO/TQFCR).
